# Glycogen Synthase Kinase 3β (GSK3β) Regulates Myogenic Differentiation in Skeletal Muscle Satellite Cells of Sheep

**DOI:** 10.3390/ani12202789

**Published:** 2022-10-15

**Authors:** Jingquan Yang, Haosen Yang, Linjie Wang, Ping Zhou

**Affiliations:** 1 State Key Laboratory of Sheep Genetic Improvement and Healthy Production, Xinjiang Academy of Agricultural and Reclamation Sciences, Shihezi 832000, China; 2 College of Animal Science and Technology, Sichuan Agricultural University, Chengdu 611130, China

**Keywords:** sheep, GSK3β, skeletal muscle, satellite cells, SB216763

## Abstract

**Simple Summary:**

In this study, we investigated the function of GSK3β in the skeletal muscle satellite cells (SMSCs) of sheep. The overexpression of *GSK3β* inhibited myotube formation and the expression of *MyoD*, *MyoG*, *MyHC1*, and *MyHC2a* genes in sheep SMSCs. Additionally, inhibiting the activity of GSK3β significantly promoted myotube formation as well as *MyoD*, *MyoG*, *MyHC1*, and *MyHC2a* genes at mRNA levels. The present study provides evidence for studying the mechanisms involved in the regulation of sheep SMSCs differentiation by GSK3β.

**Abstract:**

Glycogen synthase kinase 3β (GSK3β) has a vital role in the regulation of many cellular processes. However, the role of GSK3β in muscle cell differentiation in sheep remains unknown. In this study, we investigated the function of GSK3β in skeletal muscle satellite cells (SMSCs) of sheep. An overexpression of *GSK3β* significantly inhibited myotube formation as well as the mRNA levels of myogenic genes (*MyoD*, *MyoG*, *MyHC1*, and *MyHC2a*) in sheep SMSCs. SB216763 treatment had a time-course effect on the phosphorylation levels of sheep GSK3β. In addition, reducing the activity of GSK3β lead to the promotion of sheep SMSCs differentiation as well as the mRNA levels of myogenic genes (*MyoD*, *MyoG*, *MyHC1*, and *MyHC2a*). This study illustrated the function of GSK3β to inhibit myogenesis in sheep SMSCs, which provided evidence for studying the mechanisms involved in the regulation of sheep SMSCs differentiation by GSK3β.

## 1. Introduction

Glycogen synthase kinase 3β (GSK3β) was originally known as a vital enzyme in glycogen metabolism biosynthesis [1,2]. Glycogen Synthase (GS) is an enzyme that is involved in converting glucose to glycogen. Serine 9 phosphorylation of GSK3β leads to a loss of GSK3 catalytic activity [3]. It is well accepted that GSK3β acts as a key and negative regulatory kinase of GS. IGF-1 can regulate the GSK3β activity through the phosphorylation regulation of GSK3β, and GS is the direct substrate of GSK3β. With further study on GSK3β, it was demonstrated that GSK3β is not only an enzyme in glycogen metabolism biosynthesis but also an important regulator of many cell signaling pathways [4]. In mice, GSK3β phosphorylates PPARα at the Ser73 site, thereby inhibiting PPARα activity. This leads to elevate blood glucose levels and severe liver steatosis [5]. Additionally, GSK3β reduces brown adipocyte thermogenesis by inhibiting MAPK to regulate thermogenic gene expression [6]. GSK3β promotes the differentiation of human adipose-derived stem cells, suggesting its potential to regulate stem cell differentiation [7]. Furthermore, a knockdown of GSK3β induces the formation of multiple axons in neurons, whereas the overexpression of *GSK3β* in neurons inhibits axon arborization [8]. These studies demonstrate that GSK3β regulates cell differentiation and metabolism. 

Skeletal muscle originates from the mesoderm, and its generation is initiated within the soma by pre-muscular progenitors and skeletal myoblasts. The embryonic period is dominated by the PAX7-mediated proliferation of muscle progenitors and muscle cell fusion, whereas the postnatal differentiation of predominantly skeletal muscle satellite cells (SMSCs) adds new myofibril results into the hypertrophy of individual muscle fibers [9]. Skeletal muscle is the largest tissue in livestock, comprising 35–60% body weight [10]. Skeletal muscle satellite cells are a type of muscle-derived stem cell with proliferative and differentiation potential that are normally quiescent in adult animals. When stimulated by exercise or muscle injury, satellite cells are activated and enter mitosis, where they undergo cell division and give rise to myogenic progenitors, promoting muscle regeneration [11]. Additionally, skeletal muscle satellite cells have myogenic stem cell potential and are activated into muscle cells, while myoblasts differentiate into myotubes under specific conditions (such as skeletal muscle injury) [11]. The development of skeletal muscle and the differentiation of SMSCs is regulated by multiple transcription factors [12]. Among them, *MyOD*, *MyOG*, and *MyHC* genes regulate myotube formation and are markers of SMSCs differentiation. [13]. 

GSK3β has an important regulatory role in satellite cell differentiation and development. GSK3β knockout in mice leads to hypertrophic cardiomyopathy caused by excessive cardiomyocyte proliferation [14]. GSK3β regulates MEF2 activity indirectly by regulating the p38/MAPK pathway, and cardiac-specific GSK3β knockout mice result in the upregulation of p38/MAPK activity [15]. IGF-I induces the phosphorylation of GSK3β, and then the phosphorylation of GSK3β promotes MRF expression and muscle regeneration [16]. A sepsis-induced increase in muscle proteolysis can be effectively reversed by the mTOR signaling pathway through the inhibition of GSK3β activity [17]. Low-dose lithium supplementation enhances the muscle antifatigue capacity in mice by inhibiting GSK3β [18]. The inhibition of GSK3β can increase the transcriptional activity of MYHC2a [19], probably by phosphorylating NFAT and inhibiting its gene transcription in response to MYHC2a [20]. In addition, GSK3β is important for the regulation of muscle hypertrophy versus atrophy. Myotube atrophy and myofibrillar protein loss are dependent on GSK3β, and the inhibition of GSK3β leads to skeletal myotube hypertrophy [21,22]. In addition, GSK3β promotes myogenic differentiation and myoblast fusion through the Wnt/β-Catenin signaling pathway [23]. These studies suggest that GSK3β may have an important regulatory role for muscle development.

Skeletal muscle development is important in improving meat production in farm animals. The muscle development of sheep directly affects meat production of sheep [24]. GSK3β exhibits a strong regulatory function for muscle cell differentiation. Our previous study identified multiple and alternative forms of splicing and differential expression patterns in the *GSK3β* gene [25]. In addition, the inhibition of GSK3β increased the binding ability of PPARγ to the *NAMPT* promoter of goat adipocytes and promoted the expression of the *NAMPT* gene [26]. However, the role of GSK3β in the regulation of muscle cell differentiation in sheep remains unknown. Therefore, it is important to understand the regulation of GSK3β in sheep SMSCs. In this study, we investigated the function of GSK3β in the SMSCs of sheep. Gain-of-function experiments demonstrated that an overexpression of *GSK3β* inhibited myotube formation and the expression of myogenic genes (*MyoD*, *MyoG*, *MyHC1*, and *MyHC2a*) in sheep SMSCs. Use of SB216763 to inhibit GSK3β activity significantly promoted myotube formation and increased the mRNA levels of key myogenic genes (*MyoD*, *MyoG*, *MyHC1*, and *MyHC2a*). This study illustrates the ability of GSK3β to inhibit myogenesis in sheep SMSCs, which provides evidence for investigating the mechanism by which GSK3β regulates the differentiation of sheep SMSCs.

## 2. Materials and Methods

### 2.1. Ethics Approval

All research involving animals was conducted according to the approved protocols of the Institutional Animal Care and Use Committee at the College of Animal Science and Technology, Sichuan Agricultural University, Sichuan, China, under the ethics approval number: DKY-202000551. 

### 2.2. Sheep SMSCs Isolation

Satellite cells were isolated from the *longissimus dorsi* of three Liangshan semi-fine wool sheep on day 1 after birth using the pronase digestion method. Skeletal muscles were excised and digested with 0.2% pronase and then placed in 37 °C water for 60 min. Then, cells were separated from tissue fragments by centrifugation at 1000× *g* for 5 min, followed by filtration through a 200 μm and 50 μm Nytex filters. Sheep SMSCs were cultured in DMEM and supplemented with 15% FBS at 37 °C with 5% CO_2_. For myogenic differentiation, cells were induced with DMEM and 2% horse serum for 8 days. To caculate the fusion index, the number of fusion nuclei (two or more nuclei per cell) was counted. The fusion index (%) is the ratio of fusional nuclei to the total cell nuclei.

### 2.3. Plasmid Construct and Transfection

The ORF of the *GSK3β* gene was ligated to the Hind III and BamH I sites of the pcDNA3.1(+) by T4 DNA Ligase (TaKaRa, Dalian, China). After sequencing, the plasmid constructs (pcDNA3.1-*GSK3β*) were obtained by the Endo-free Plasmid Mini Kit II (Omega Bio-Tek, Norcross, GA, USA). After cells were grown to 80% confluence, a 4 μg recombinant vector pcDNA3.1-GSK3β with 7.5 μL Lipofectamine™ 3000 (Invitrogen, Carlsbad, CA, USA) were transfected into the cells of a 35 mm cell culture dish.

### 2.4. Cell Culture and GSK3β Inhibitor Treatment

To evaluate the role of time-course SB216763 in the regulation of mRNA expression patterns of GSK3β, when 90% confluence was observed, muscle satellite cells were serum starved for 6 h, then 10 μM SB216763 was added for 0, 2, 4, and 8 days during myogenic differentiation. 

### 2.5. Quantitative PCR (qPCR)

Cells in six-well plate were harvested from each group on day 8 of differentiation (*n* = 6) for qPCR analysis. The total RNA was extracted by RNAiso plus (Takara, Tokyo, Japan). RNA concentration was quantified at 260 nm by a nucleic acid protein detector (Bio-Rad, Hercules, CA, USA). An amount of 1 μg total RNA was used to synthesize cDNA using a PrimeScript RT reagent kit. qPCR was conducted by a SYBR GreenⅡqPCR kit (Takara, Tokyo, Japan). The 2^−ΔΔCt^ method was adopted to calculate the relative gene expression level normalized to *GAPDH*. Primers are shown in Table S1.

### 2.6. Western Blotting

The total protein of the skeletal muscle satellite cells was extracted by a protein extraction kit. The content of protein was determined by a BCA protein quantification detection kit (Google Biotechnology, Wuhan, China) which was then separated by SDS-PAGE and transferred to a PVDF membrane following immunoblotting with a primary antibody against Ser9-GSK3β (1:400, Santa Cruz, CA, USA). This antibody was used to determine the phosphorylation level of Ser9-GSK3β and phosphorylation levels of Ser9-GSK3β when normalized to GAPDH. The PVDF membrane was incubated with the HRP-labeled goat anti-rabbit IgG (1:2000, Santa Cruz, CA, USA) for two hours and visualized by an ECL chemiluminescence system. 

## 3. Results

### 3.1. Overexpression of GSK3β Decreases Differentiation in Sheep SMSCs

**Figure 1:**
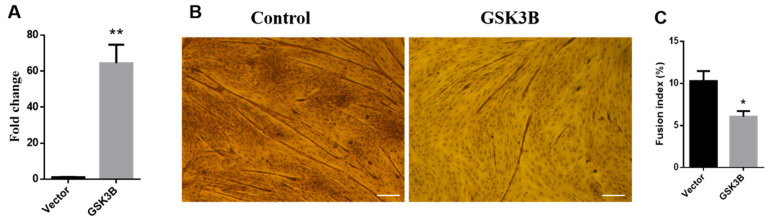
GSK3β decreases myogenic differentiation in sheep SMSCs. (**A**) The expression level of *GSK3β* in sheep SMSCs. Cells were transfected with the *GSK3β* overexpression vector (pcDNA3.1-*GSK3β*). (**B**) Myotube formation was visualized by an inverted light microscope and (**C**) quantified by measuring the fusion index at 8 days of differentiation. The fusion index (%) is the ratio of fusional nuclei to the total cell nuclei within the same field of vision. *p* < 0.05 (*) and *p* < 0.01 (**) relative to control (pcDNA3.1+).

To evaluate the effective overexpression of the *GSK3β* gene, we determined the mRNA expression level of the sheep *GSK3β* gene. Compared with the control group, the *GSK3β* gene expression in muscle satellite cells transfected with the pcDNA3.1-*GSK3β* vector was overexpressed roughly 60-fold, indicating that the pcDNA3.1-*GSK3β* vector was successfully transfected into muscle satellite cells and promoted the mRNA expression of the sheep *GSK3β* gene (*p* < 0.01) (Figure 1A). 

**Figure 2:**
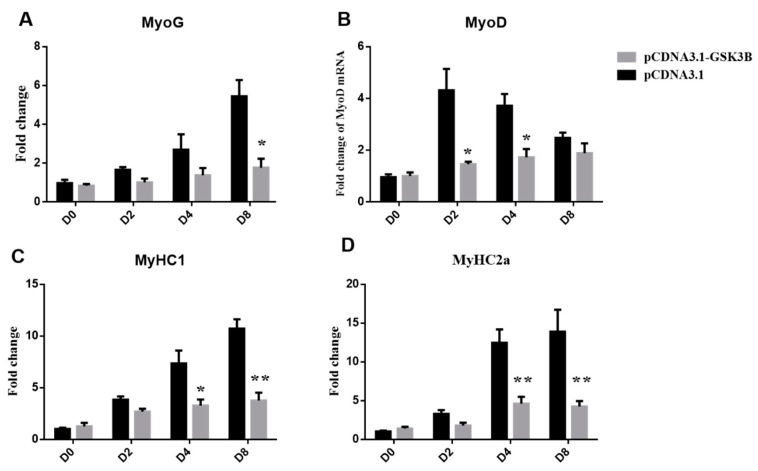
GSK3β decreases expression levels of myogenic genes in sheep SMSCs. (**A**–**D**) The expression levels of *MyoG*, *MyoD*, *MyHC1*, and *MyHC2a* genes. All data are expressed as mean ± S.E.M. (*n* = 6). *p* < 0.05 (*) and *p* < 0.01 (**) relative to control (pcDNA3.1+).

Moreover, *GSK3β* overexpression strongly decreased the myogenic differentiation of sheep skeletal muscle satellite cells (Figure 1B). GSK3β inhibited myotube formation, and the fusion index was significantly decreased at 8 days of differentiation compared with the control (*p* < 0.05) (Figure 1C). *GSK3β* overexpression significantly decreased expression levels of MyoG at 8 days of differentiation (*p* < 0.05) (Figure 2A). In addition, *GSK3β* overexpression robustly impaired expression of the *MyoD* gene at 2 and 4 days of differentiation (*p* < 0.05) (Figure 2B). Additionally, expression levels of *MyHC1* and *MyHC2a* were significantly decreased at 4 and 8 days of differentiation (*p* < 0.01) during sheep SMSCs differentiation. (Figure 2C,D). These results suggest that an overexpression of *GSK3β* can inhibit the differentiation and key myogenic gene expression of sheep SMSCs. 

### 3.2. Different Effects of Time-Course SB216763 Treatment on the Phosphorylation Levels of GSK3β in Sheep SMSCs

**Figure 3:**
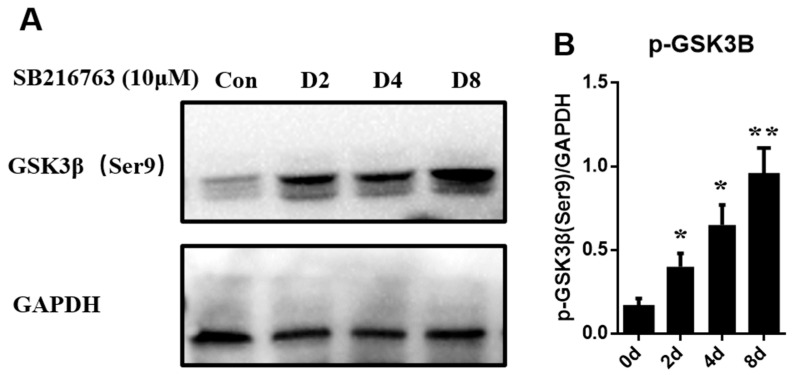
Effects of time-course SB216763 treatment on the phosphorylation levels of GSK3β in sheep SMSCs. Cells were serum starved for 6 h, then SB216763 (10 μM) was added for 0, 2, 4, and 8 days. (**A**) Phosphorylation of GSK3β (Ser9) was induced by SB216763 in sheep skeletal muscle satellite cells. (**B**) Quantified results of western blots. *p* < 0.05 (*) and *p* < 0.01 (**) relative to 0d of differentiation.

SB216763 is an effective small molecule inhibitor and belongs to maleimide. It can promote the phosphorylation of GSK3β ser9 to inhibit the activity of GSK3β. We identified the role of SB216763 in the regulation of the phosphorylation levels of sheep GSK3β. As shown in Figure 3, SB216763 treatment had a time-course effect on the phosphorylation levels of sheep GSK3β. After the SB216763 treatment, the phosphorylation levels of sheep GSK3β were significantly upregulated (*p* < 0.05) at 2 days and reached the highest level at 8 days of differentiation (*p* < 0.01). These results suggest that SB216763 increases the phosphorylation of GSK3β during sheep SMSCs differentiation. 

### 3.3. GSK3β Inhibition Promotes Differentiation of Sheep SMSCs 

**Figure 4:**
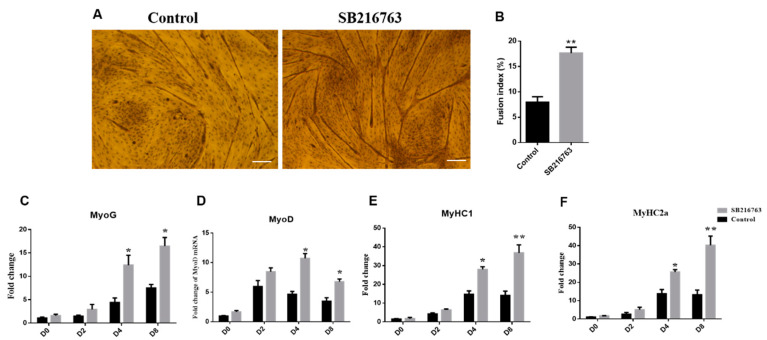
GSK3β inhibition promoted myotube formation of sheep skeletal muscle satellite cells. (**A**) Myotube formation was visualized by an inverted light microscope and (**B**) quantified by measuring the fusion index at 8 days of differentiation after SB216763 (10 μM) treatment for 8 days. The fusion index (%) is the ratio of fusional nuclei to the total cell nuclei within the same field of vision. (**C**–**F**) The expression levels of *MyoG*, *MyoD*, *MyHC1*, and *MyHC2a* genes. All data are expressed as mean ± S.E.M. (*n* = 6). *p* < 0.05 (*) and *p* < 0.01 (**) relative to control.

As shown in Figure 4A, GSK3β inhibition by SB216763 strongly promoted the myotube formation of sheep SMSCs. The fusion index was significantly increased at D8 differentiation compared with the control (*p* < 0.01) (Figure 4B). GSK3β inhibition significantly increased expression levels of *MyoG* at 4 and 8 days of differentiation (*p* < 0.05) (Figure 4C). Additionally, inhibiting activities of GSK3β were shown to robustly upregulate the expression levels of the *MyoD* gene at 4 and 8 days of differentiation (*p* < 0.05) (Figure 4D). In addition, the expression levels of *MyHC1* and *MyHC2a* were significantly increased at 4 and 8 days of differentiation (*p* < 0.01). (Figure 4E,F). These results suggest that the inhibiting GSK3β activity promotes myotube formation and the expression of key myogenic genes in SMSCs. 

## 4. Discussion

GSK3β belongs to the serine-threonine kinases that regulate glucose homeostasis. GSK3β can phosphorylate over a hundred substrates, and its phosphorylation at the Ser9 site can lead to its inactivation [27]. There are two isoforms of GSK3, of which GSK3β is the most highly expressed and active isoform in skeletal muscle. GSK3β is a key regulator enzyme in glycogen synthesis, and it can inhibit GS activity by phosphorylating GS, thereby inhibiting glycogen synthesis [28]. In type 2 diabetes (T2D), the insulin-mediated decrease in GS activity and glycogen synthesis is closely associated with an increase in GSK3β levels in the muscle [29]. There is a large population of resident mesenchymal stem cells in fetal muscle tissue, which differentiate into mature adipocytes. In addition, several molecules that regulate the adipogenic or myogenic differentiation fates of cells were identified. For example, a knockdown of EHMT1 in adipocytes leads to the demethylation of histone 3 lysine 9 (H3K9me2 and 3), inducing muscle differentiation in vivo [30]. Furthermore, the loss of PRDM16 in brown preadipocytes leads to impaired brown adipocyte differentiation and promotes muscle differentiation [31]. Our previous study showed that GSK3β promoted the adipogenic differentiation of goat skeletal muscle satellite cells by activating the AMPK pathway. Meanwhile, inhibition of GSK3β resulted in a significant downregulation in the expression of adipogenic differentiation marker genes [32]. In the present study, GSK3β played a critical role in the differentiation of myoblasts in sheep. Gain-of-function experiments demonstrated that GSK3β inhibited myotube formation in sheep SMSCs. Inhibiting the activity of GSK3β significantly promoted myotube formation. These results demonstrate that GSK3β is important for sheep SMSCs myogenic differentiation. Furthermore, the distinct regulatory effects of GSK3β on the adipogenic and myogenic differentiation of sheep SMSCs suggest its possible involvement in sheep SMSCs and differentiation fate commitment.

Previous studies have demonstrated that skeletal muscle GSK3β knockout mice have increased myonuclear proliferation in their regeneration of skeletal muscle [33]. GSK3β can be phosphorylated through PI3-K/Akt in response to the insulin signaling pathway before decreasing the transcription factor FOXO1 activity, thereby affecting mitogenesis in C2C12 cells [34]. Meanwhile, the GSK3β gene is inactivated by the PI3K/Akt pathway, which increased the expression of muscle cell differentiation marker genes and promoted muscle cell differentiation [16,35]. An inhibition of GSK3β can activate the β-Catenin-TCF complex to promote human muscle progenitor differentiation, which is necessary for their cellular differentiation [36]. Previous studies have reported a significant enrichment of MyoD on day 2 of sheep SMSCs differentiation, while MyoG appeared significantly enriched after day 4 of differentiation [37]. In this study, the overexpression of *GSK3β* decreased the expression of *MyoD*, *MyoG*, *MyHC1*, and *MyHC2a* genes in sheep SMSCs after day 4 of differentiation. These results indicate that GSK3β negatively regulates myogenic gene expression and may play a regulatory role after day 4 of differentiation. 

Previous studies show that SB216763 is an effective small molecule inhibitor and belongs to maleimide. It increases the phosphorylation of GSK3β Ser9 by competing with ATP [38,39]. SB216763 protects against aldosterone-induced cardiac and renal injury by inhibiting the activity of GSK3β [40]. SB216763 inhibits the activity of GSK3β by increasing Ser9 phosphorylation, thereby inhibiting the proliferation and migration of squamous cancer cells [41]. Furthermore, the inhibition of GSK3β by SB216763 prevents cardiomyocyte apoptosis by increasing the Ser9 phosphorylation of GSK3β [42]. SB216763 inhibits the activity of GSK3β and reduces the nuclear activity of the NFKB1 pathway, which alleviates neuritis [43]. The chondrogenic differentiation of human MSCs, by adding SB216763 to inhibit GSK3β, significantly enhanced cartilage matrix production and the expression of cartilage-specific genes [44]. These studies suggest that SB216763 is an effective inhibitor when studying the function of GSK3β. In this study, the treatment of SB216763 in sheep SMSCs increased the Ser9 phosphorylation of GSK3β at 2 days and reached the highest level at 8 days of differentiation, which inhibited GSK3β activity. Additionally, inhibiting the activity of GSK3β increased the mRNA levels of myogenic genes after day 4 of differentiation. These results illustrate that GSKβ3 inhibition promoted sheep SMSC myogenic differentiation after day 4 of differentiation.

When skeletal muscle satellite cells are induced to myogenic differentiation in vitro, the pre-differentiation phase is myotube formation mediated by MyoD, whereas the post-differentiation phase is MyoG-mediated maturation and a confluence of myotubes [45]. Yang et al. found that the MyoD protein is mainly expressed during the first 24 h of skeletal muscle satellite cell differentiation, whereas the MyoG protein is mainly expressed after day 1 of skeletal muscle satellite cell differentiation [37]. These studies suggest that myogenic transcription factors are expressed at specific times of differentiation. In the present study, *GSK3β* overexpression significantly decreased expression levels of *MyoG* at 8 days of differentiation. In addition, *GSK3β* overexpression robustly impaired expression levels of the *MyoD* gene at 2 and 4 days of differentiation. The regulation of myogenic transcription factors by an overexpression of *GSK3β* is in agreement with previous studies. However, SB216763 inhibited the activities of GSK3β and significantly increased the expression levels of *MyoG* at 4 and 8 days of differentiation. Additionally, GSK3β inhibition robustly upregulated expression levels of the *MyoD* gene at 4 and 8 days of differentiation. The inhibition of GSK3β, which mainly regulates myogenic transcription factors at the post-stage of skeletal muscle satellite cells differentiation, still needs to be investigated. 

## 5. Conclusions

In this study, we have identified the roles of GSK3β in sheep SMSCs differentiation. The overexpression of *GSK3β* inhibited the differentiation of sheep SMSCs and decreased the expression of key myogenic genes in SMSCs. In addition, SB216763 treatment had a time-course effect on the phosphorylation levels of GSK3β, suggesting that SB216763 is an effective inhibitor for GSK3β in sheep SMSCs. Furthermore, inhibiting the activity of GSK3β promoted sheep SMSCs differentiation and increased the mRNA levels of myogenic genes after day 4 of differentiation. In conclusion, the present study indicates the function of GSK3β to inhibit myogenesis in sheep SMSCs. 

## Data Availability

The data presented in this study are available on request from the corresponding author.

## Supplementary Materials

The following supporting information can be downloaded at: https://www.mdpi.com/article/10.3390/ani12202789/s1, Table S1. Primer sequences used in this study.

## References

[1] Woodgett J.R., Cohen P. (1984). Multisite phosphorylation of glycogen synthase. Molecular basis for the substrate specificity of glycogen synthase kinase-3 and casein kinase-II (glycogen synthase kinase-5). Biochim. Biophys. Acta.

[2] Rylatt D.B., Aitken A., Bilham T., Condon G.D., Embi N., Cohen P. (1980). Glycogen synthase from rabbit skeletal muscle. Amino acid sequence at the sites phosphorylated by glycogen synthase kinase-3, and extension of the N-terminal sequence containing the site phosphorylated by phosphorylase kinase. Eur. J. Biochem..

[3] Beurel E., Grieco S.F., Jope R.S. (2015). Glycogen synthase kinase-3 (GSK3): Regulation, actions, and diseases. Pharmacol. Ther..

[4] Cortes-Vieyra R., Bravo-Patino A., Valdez-Alarcon J.J., Juarez M.C., Finlay B.B., Baizabal-Aguirre V.M. (2012). Role of glycogen synthase kinase-3 beta in the inflammatory response caused by bacterial pathogens. J. Inflamm..

[5] Hinds T.D., Burns K.A., Hosick P.A., McBeth L., Nestor-Kalinoski A., Drummond H.A., AlAmodi A.A., Hankins M.W., Vanden Heuvel J.P., Stec D.E. (2016). Biliverdin Reductase A Attenuates Hepatic Steatosis by Inhibition of Glycogen Synthase Kinase (GSK) 3β Phosphorylation of Serine 73 of Peroxisome Proliferator-activated Receptor (PPAR) α. J. Biol. Chem..

[6] Markussen L.K., Winther S., Wicksteed B., Hansen J.B. (2018). GSK3 is a negative regulator of the thermogenic program in brown adipocytes. Sci. Rep..

[7] Zaragosi L.E., Wdziekonski B., Fontaine C., Villageois P., Peraldi P., Dani C. (2008). Effects of GSK3 inhibitors on in vitro expansion and differentiation of human adipose-derived stem cells into adipocytes. BMC Cell Biol..

[8] Jiang H., Guo W., Liang X., Rao Y. (2005). Both the establishment and the maintenance of neuronal polarity require active mechanisms: Critical roles of GSK-3beta and its upstream regulators. Cell.

[9] Chal J., Pourquié O. (2017). Making muscle: Skeletal myogenesis in vivo and in vitro. Development.

[10] Berendsen A.D., Olsen B.R. (2015). Bone development. Bone.

[11] Chang N.C., Rudnicki M.A. (2014). Satellite cells: The architects of skeletal muscle. Curr. Top. Dev. Biol..

[12] Li J., Pei Y., Zhou R., Tang Z., Yang Y. (2021). Regulation of RNA N(6)-methyladenosine modification and its emerging roles in skeletal muscle development. Int. J. Biol. Sci..

[13] Yin H., Price F. (2013). Rudnicki, M.A. Satellite cells and the muscle stem cell niche. Physiol. Rev..

[14] Kerkela R., Kockeritz L., Macaulay K., Zhou J., Doble B.W., Beahm C., Greytak S., Woulfe K., Trivedi C.M., Woodgett J.R. (2008). Deletion of GSK-3beta in mice leads to hypertrophic cardiomyopathy secondary to cardiomyoblast hyperproliferation. J. Clin. Investig..

[15] Dionyssiou M.G., Nowacki N.B., Hashemi S., Zhao J., Kerr A., Tsushima R.G., McDermott J.C. (2013). Cross-talk between glycogen synthase kinase 3β (GSK3β) and p38MAPK regulates myocyte enhancer factor 2 (MEF2) activity in skeletal and cardiac muscle. J. Mol. Cell. Cardiol..

[16] Vyas D.R., Spangenburg E.E., Abraha T.W., Childs T.E., Booth F.W. (2002). GSK-3beta negatively regulates skeletal myotube hypertrophy. Am. J. Physiol. Cell Physiol..

[17] Bertsch S., Lang C.H., Vary T.C. (2011). Inhibition of glycogen synthase kinase 3[beta] activity with lithium in vitro attenuates sepsis-induced changes in muscle protein turnover. Shock.

[18] Whitley K.C., Hamstra S.I., Baranowski R.W., Watson C.J.F., MacPherson R.E.K., MacNeil A.J., Roy B.D., Vandenboom R., Fajardo V.A. (2020). GSK3 inhibition with low dose lithium supplementation augments murine muscle fatigue resistance and specific force production. Physiol. Rep..

[19] Wang L., Zhu Y., Liu X., Chao Z., Wang Y., Zhong T., Guo J., Zhan S., Li L., Zhang H. (2019). Glycogen synthase kinase 3β (GSK3β) regulates the expression of MyHC2a in goat skeletal muscle satellite cells (SMSCs). Anim. Sci. J. Nihon Chikusan Gakkaiho.

[20] Jiang H., Li H., DiMario J.X. (2006). Control of slow myosin heavy chain 2 gene expression by glycogen synthase kinase activity in skeletal muscle fibers. Cell Tissue Res..

[21] Verhees K.J., Schols A.M., Kelders M.C., Op den Kamp C.M., van der Velden J.L., Langen R.C. (2011). Glycogen synthase kinase-3β is required for the induction of skeletal muscle atrophy. Am. J. Physiol. Cell Physiol..

[22] van der Velden J.L., Langen R.C., Kelders M.C., Willems J., Wouters E.F., Janssen-Heininger Y.M., Schols A.M. (2007). Myogenic differentiation during regrowth of atrophied skeletal muscle is associated with inactivation of GSK-3beta. Am. J. Physiol. Cell Physiol..

[23] Pansters N.A., van der Velden J.L., Kelders M.C., Laeremans H., Schols A.M., Langen R.C. (2011). Segregation of myoblast fusion and muscle-specific gene expression by distinct ligand-dependent inactivation of GSK-3β. Cell. Mol. Life Sci..

[24] Prache S., Schreurs N., Guillier L. (2022). Review: Factors affecting sheep carcass and meat quality attributes. Anim. Int. J. Anim. Biosci..

[25] Hou Y., Wang Y., Wang Y., Zhong T., Li L., Zhang H., Wang L. (2014). Multiple alternative splicing and differential expression pattern of the glycogen synthase kinase-3β (GSK3β) gene in goat (Capra hircus). PLoS ONE.

[26] Wang L., Li X., Chao Z., Zhong T., Guo J., Wang Y., Li L., Zhang H. (2019). Transcriptional Regulation of NAMPT Gene by Glycogen Synthase Kinase 3β in Goat Adipocytes. DNA Cell Biol..

[27] Wang L., Zuo B., Xu D., Ren Z., Zhang H., Li X., Lei M., Xiong Y. (2012). Alternative splicing of the porcine glycogen synthase kinase 3beta (GSK-3beta) gene with differential expression patterns and regulatory functions. PLoS ONE.

[28] Ring D.B., Johnson K.W., Henriksen E.J., Nuss J.M., Goff D., Kinnick T.R., Ma S.T., Reeder J.W., Samuels I., Slabiak T. (2003). Selective glycogen synthase kinase 3 inhibitors potentiate insulin activation of glucose transport and utilization in vitro and in vivo. Diabetes.

[29] Nikoulina S.E., Ciaraldi T.P., Mudaliar S., Carter L., Johnson K., Henry R.R. (2002). Inhibition of glycogen synthase kinase 3 improves insulin action and glucose metabolism in human skeletal muscle. Diabetes.

[30] Ohno H., Shinoda K., Ohyama K., Sharp L.Z., Kajimura S. (2013). EHMT1 controls brown adipose cell fate and thermogenesis through the PRDM16 complex. Nature.

[31] Seale P., Bjork B., Yang W., Kajimura S., Chin S., Kuang S., Scimè A., Devarakonda S., Conroe H.M., Erdjument-Bromage H. (2008). PRDM16 controls a brown fat/skeletal muscle switch. Nature.

[32] Wang L., Liu X., Zhan S., Guo J., Yang S., Zhong T., Li L., Zhang H., Wang Y. (2019). Inhibition of GSK3β Reduces Ectopic Lipid Accumulation and Induces Autophagy by the AMPK Pathway in Goat Muscle Satellite Cells. Cells.

[33] Pansters N.A., Schols A.M., Verhees K.J., de Theije C.C., Snepvangers F.J., Kelders M.C., Ubags N.D., Haegens A., Langen R.C. (2015). Muscle-specific GSK-3β ablation accelerates regeneration of disuse-atrophied skeletal muscle. Biochim. Biophys. Acta.

[34] Litwiniuk A., Pijet B., Pijet-Kucicka M., Gajewska M., Pająk B., Orzechowski A. (2016). FOXO1 and GSK-3β Are Main Targets of Insulin-Mediated Myogenesis in C2C12 Muscle Cells. PLoS ONE.

[35] van der Velden J.L., Langen R.C., Kelders M.C., Wouters E.F., Janssen-Heininger Y.M., Schols A.M. (2006). Inhibition of glycogen synthase kinase-3beta activity is sufficient to stimulate myogenic differentiation. Am. J. Physiol.-Cell Physiol..

[36] Agley C.C., Lewis F.C., Jaka O., Lazarus N.R., Velloso C., Francis-West P., Ellison-Hughes G.M., Harridge S.D.R. (2017). Active GSK3β and an intact β-catenin TCF complex are essential for the differentiation of human myogenic progenitor cells. Sci. Rep..

[37] Yang J.H., Chang M.W., Pandey P.R., Tsitsipatis D., Yang X., Martindale J.L., Munk R., De S., Abdelmohsen K., Gorospe M. (2020). Interaction of OIP5-AS1 with MEF2C mRNA promotes myogenic gene expression. Nucleic Acids Res..

[38] Wang L., Li X., Wang Y. (2018). GSK3beta inhibition attenuates LPS-induced IL-6 expression in porcine adipocytes. Sci. Rep..

[39] Westmark P.R., Garrone B., Ombrato R., Milanese C., Di Giorgio F.P., Westmark C.J. (2021). Testing Fmr1 (KO) Phenotypes in Response to GSK3 Inhibitors: SB216763 versus AFC03127. Front. Mol. Neurosci..

[40] Zhang Y.D., Ding X.J., Dai H.Y., Peng W.S., Guo N.F., Zhang Y., Zhou Q.L., Chen X.L. (2018). SB-216763, a GSK-3β inhibitor, protects against aldosterone-induced cardiac, and renal injury by activating autophagy. J. Cell. Biochem..

[41] Schulz L., Pries R., Lanka A.S., Drenckhan M., Rades D., Wollenberg B. (2018). Inhibition of GSK3α/β impairs the progression of HNSCC. Oncotarget.

[42] Wang W., Yang Y., Xiong Z., Kong J., Fu X., Shen F., Huang Z. (2016). Inhibition of glycogen synthase kinase 3beta ameliorates triptolide-induced acute cardiac injury by desensitizing mitochondrial permeability transition. Toxicol. Appl. Pharmacol..

[43] Jorge-Torres O.C., Szczesna K., Roa L., Casal C., Gonzalez-Somermeyer L., Soler M., Velasco C.D., Martínez-San Segundo P., Petazzi P., Sáez M.A. (2018). Inhibition of Gsk3b Reduces Nfkb1 Signaling and Rescues Synaptic Activity to Improve the Rett Syndrome Phenotype in Mecp2-Knockout Mice. Cell Rep..

[44] Baghaban Eslaminejad M., Karimi N., Shahhoseini M. (2011). Enhancement of Glycosaminoglycan-Rich Matrix Production in Human Marrow-Derived Mesenchymal Stem Cell Chondrogenic Culture by Lithium Chloride and SB216763 Treatment. Cell J..

[45] Romagnoli C., Iantomasi T., Brandi M.L. (2021). Available In Vitro Models for Human Satellite Cells from Skeletal Muscle. Int. J. Mol. Sci..

