# Supplementary material for: Glycogen Synthase Kinase 3β (GSK3β) Regulates Myogenic Differentiation in Skeletal Muscle Satellite Cells of Sheep
Source: Animals (Basel). 2022 Oct 15;12(20):2789. doi: 10.3390/ani12202789 (PMC9597728; doi:10.3390/ani12202789)
Supplement: Supplementary file 1 [file animals-12-02789-s001.zip › animals-1900142-supplementary.pdf]

Table S1: Primer sequences used in this study.

Gene name	Primer sequence (5′–3′)	Tm (°C)
GSK3B	ATGTCAGGGCGGCCCAGAA	63
	TCAGGAGGAGTCGGAAGC	
MyHC2a	TTTGGGGAGGCTGCTCCTTA	61
	AAAGATTCCTTGGGCTCGGC	
MyHC1	GAGCTCACGTACCAGACAGAG	60
	CAGACCAAGAAGACGTGGCA	
MyoD	GCTCCAGAACCGCAGTAAGTT	61
	CGGCGACAGCAGCTCCATA	
MyoG	GAAGCGCAGACTCAAGAAGG	61
	TGCAGGCGCTCTATGTACTG	
GAPDH	GGTCGGAGTGAACGGATTTG	60
	TGGCAACGATGTCCACTTTG	
